# Bridging the Vaccination Equity Gap: A Community-Driven Approach to Reduce Vaccine Inequities in Polio High-Risk Areas of Pakistan

**DOI:** 10.3390/vaccines12121340

**Published:** 2024-11-28

**Authors:** Imran A. Chauhadry, Sajid Bashir Soofi, Muhammad Sajid, Rafey Ali, Ahmad Khan, Syeda Kanza Naqvi, Imtiaz Hussain, Muhammad Umer, Zulfiqar A. Bhutta

**Affiliations:** 1 Centre of Excellence in Women and Child Health, Aga Khan University, Karachi 74800, Pakistan; imran.ahmed@aku.edu (I.A.C.); sajid.soofi@aku.edu (S.B.S.); ahmad.sherali@aku.edu (A.K.); imtiaz.hussain@aku.edu (I.H.); muhammad.umer@aku.edu (M.U.); 2 Department of Pediatrics & Child Health, Aga Khan University, Karachi 74800, Pakistan; 3 Institute for Global Health and Development, Aga Khan University, Karachi 74800, Pakistan; 4 Centre for Global Child Health, The Hospital for Sick Children, Toronto, ON M5G 1X8, Canada

**Keywords:** vaccination equity, wealth-based disparities, community engagement, polio high-risk areas, Pakistan

## Abstract

**Background:** Immunization saves millions of lives, and globally, vaccines have significantly contributed to reducing mortality and morbidity due to more than 20 life-threatening illnesses. However, there are considerable disparities in vaccination coverage among countries and within populations. This study evaluates the reduction in disparities in vaccination coverage across various socio-economic groups by adopting an integrated community-engagement approach combined with maternal and child health services through mobile health camps. **Methods:** This secondary analysis is based on a community-based demonstration project conducted between 2014 and 2016 across 146 union councils in polio high-risk districts of Sindh, Khyber Pakhtunkhwa (KP) and Baluchistan in Pakistan. The intervention involved structured community engagement and mobile health camps providing routine immunization alongside maternal and child health services. Data were collected through cross-sectional independent surveys using the WHO two-stage cluster technique at the baseline and the endline, covering over 120,000 children under 5 years old. Four key outcome indicators were analyzed: fully vaccinated children, under-immunized children, unvaccinated children, and polio zero-dose children for equity in vaccine uptake. **Results:** The proportion of fully vaccinated children increased in the lowest wealth quintile from 28.5% (26.7%, 30.3%) at the baseline to 51.6% (49.5%, 53.8%) at the endline. In comparison, the increase in the richest quantities was 16.2% (14.0%, 18.4%) from the baseline 56.4% (54.6%, 58.2%) to the endline 72.7% (71.1%, 74.2%). Under-vaccination dropped by 10.2% (95% CI: −11.4%, −9.1%), with the poorest quintile showing an 11.8% reduction. The gap between the highest and lowest wealth quintiles in full immunization narrowed by 6.9%, from 27.9% to 21.0% at the baseline and the endline, respectively. The prevalence of zero-dose children significantly decreased across all quintiles, with the highest reduction observed in the lowest quintile of −11.3% (−13.6%, −9.1%). The difference between the highest and lowest wealth quintiles reduced from 6.2% to 3.8%. A significant reduction in polio zero-dose children was achieved, as 13.5% (95% CI: −14.8%, −12.2%), from 29.2% (95% CI: 28.0%, 30.3%) to 15.6% (14.8%, 16.5%). **Conclusions:** This study shows that integrating community engagement with maternal and child health services through health camps can significantly enhance immunization coverage and reduce wealth-based disparities in high-risk, hard-to-reach areas. The approach improved coverage for zero-dose and fully vaccinated children, suggesting a potential for scaling in regions with access issues, conflict, and vaccine hesitancy.

## 1. Introduction

Immunization is one of the most critical public health tools, saving millions of lives annually. Each year, immunization prevents approximately 3-to-5 million deaths, safeguarding communities from outbreaks of diseases that were once widespread [1]. Since 1974, vaccination has prevented 154 million deaths, including 146 million in children under 5, with 101 million being infants under the age of 1 year [2].

The disparities in immunization coverage persist, particularly in low- and middle-income countries, where 396,000 children were unvaccinated in 2023 [3]. According to the United Nations development program, 79.86% of the population living in high-income countries has received at least one dose. In comparison, this number is only 32.82% in lower-income countries. Furthermore, the healthcare cost only increases by 0.8% to cover vaccination for 70% of the population in high-income countries. On the other hand, this cost reaches up to 56.6% in low-income countries [4]. Almost 60% of the zero-dose or partially vaccinated children reside in 10 countries: Afghanistan, Angola, Ethiopia, Indonesia, India, Nigeria, Pakistan, Sudan, the Democratic Republic of Congo, and Yemen [5].

The barriers to achieving good vaccination coverage include low income, lack of parental education, poor access to health facilities, lack of trust in healthcare workers, and traditional beliefs [6]. Evidence shows a social gradient in child vaccination within countries where children belonging to well-educated, wealthy, or urban-dwelling parents are more likely to receive vaccination [7]. Global efforts to reduce disease burden can be hindered due to social inequalities in vaccination uptake in low-and middle-income countries, as children from socioeconomically disadvantaged backgrounds are more likely to acquire infectious diseases [8]. Incomplete or lack of vaccination remains a major cause of preventable child deaths in these regions [9].

Pakistan is one of the two polio-endemic countries, the other being Afghanistan, struggling to eradicate the virus. Many steps have been taken to increase polio and other routine immunization coverage by the government, which is evidenced by 86% OPV3 coverage [10]. However, disparities exist in immunization coverage across different socioeconomic groups. There is a need to assess the immunization equity gap to realize the goal of polio-free Pakistan within the global effort of polio eradication. Realizing these inequities is critical for Pakistan to achieve its immunization targets and eradication of polio. The situation is more challenging in rural areas where immunization coverage falls below 60% [11]. To counter low coverage, coordinated efforts targeting high-risk groups through integrated vaccination and maternal-child health services can improve coverage by up to 20% in some areas [12].

The study was aimed at evaluating the impact of an integrated strategy designed to enhance community engagement and maternal and child health immunization campaigns in the high-risk union councils of polio-endemic districts in Pakistan. The study involved community engagement through structured mobilization and health camps to promote vaccination and maternal and child healthcare. This strategy empowered local stakeholders, such as trained mobilizers, to take ownership of the vaccination campaigns and health-service delivery, ensuring that the interventions were culturally relevant and responsive to the community’s needs and remained effective, leading to increased acceptance and uptake of immunization services. This paper discusses findings on reducing disparities in vaccination coverage among children from various socio-economic backgrounds by integrating community-engagement efforts with maternal and child health service delivery through health camps.

## 2. Methods

### 2.1. Data Source

This analysis builds on the published community-based demonstration project between 2014 and 2016. The intervention targeted 146 union councils in Sindh, Khyber Pakhtunkhwa (KP), and Baluchistan provinces of Pakistan. The primary outcome was the change in full immunization coverage among children under 5. Secondary outcomes included coverage rates for oral polio vaccine (OPV), inactivated polio vaccine (IPV), and changes in the proportion of unvaccinated children. The intervention involved structured community mobilization led by trained mobilizers to promote vaccination and mobile health camps offering various health services, including routine immunizations and maternal and child healthcare. Detailed methodology and findings of the trial have already been published elsewhere [12].

This analysis has used data generated through cross-sectional surveys during the trial. These surveys used WHO’s 30 × 15 cluster sampling method. Fifteen households with children under 5 years of age were selected from each cluster. Parents or care providers of 122,950 children were interviewed at baseline and 133,996 at endline.

### 2.2. Outcome Indicators

Four dichotomous outcome indicators are used in this study. First, “fully vaccinated” included children who have received all age-specific doses as per the Expanded Programme of Immunization (EPI) Schedule, Pakistan starting with BCG, and OPV0 at birth, OPV 1–3, Penta 1–3, PCV 1–3, IPV, and MCV1 at the age of 9 months. The second is “under-immunized”, defined as children who have received one or more doses specific to their age, but not all. Third is “unvaccinated”, which includes children who have not received any of the scheduled vaccines through routine immunization, and lastly, polio zero dose encompassing children who have not received oral polio vaccines as part of routine immunization but have received at least one or more non-polio antigens. These definitions are context-specific and are aligned with the EPI Pakistan schedule.

### 2.3. Ethics Statement

The primary study was approved by the Ethics Review Committee of Aga Khan University, Pakistan [3307-Ped-ERC-14] and the National Bioethics Committee, Pakistan. Consent was obtained from the parents of the children who participated in the study. The trial was also registered on ClinicalTrials.gov under the identifier NCT01908114. This study is based on secondary data from this trial. Hence, no ethical concerns arise.

### 2.4. Statistical Analysis

We employed STATA version 18.0 (Stata Corp™, College Station, TX, USA) for the analysis. Stata “svy” command was used to account for the multistage stage sampling design used in the surveys. The percentages with 95% confidence intervals (CIs) were calculated for the background characteristics in both surveys.

An equity analysis has been conducted to estimate the impact of interventions on the hard-to-reach and most vulnerable segments of the population in project areas. The socio-economic status of households was assessed using a wealth index derived from household assets. In the absence of direct information on income, expenditure, and consumption, a wealth index derived from household assets serves as a reliable proxy for measuring income [13]. The wealth index was constructed as a weighted sum of various consumer durables owned by the household and other household characteristics related to wealth. The asset weights in the index were generated using principal component analysis (PCA) based on the correlation matrix of these variables. Each household was categorized into one of the five quantiles of the wealth index. We have compared the households falling in the lowest quintile with the ones in the highest quintile. The “gap” is defined as the difference between coverage in the richest and poorest quintiles at baseline against the difference between these two quintiles at endline. This coverage gap has been estimated for outcome indicators and all antigens.

The vaccination coverage stratified by different socio-demographic attributes has been presented in terms of percentages with 95% confidence intervals (CIs), and the changes in vaccine coverage between the two surveys and the *p* values were calculated using the generalized linear models (GLM).

We have also assessed the changes in the clustering of fully vaccinated and zero-dose (unvaccinated) children. We visualized the spatial distribution of immunization status across the target union councils and clusters of Karachi. We have generated two maps at the cluster level to provide a more granular analysis within each UC. Cluster-level indicators were interpolated to visualize the spatial patterns and clustering of unvaccinated and fully vaccinated children over time.

## 3. Results

The distribution of socio-demographic characteristics for the families interviewed was broadly similar between the baseline (BL) and the endline (EL). The distribution of gender and children’s age was also comparable between the two surveys. However, some improvements were observed in access to improved drinking water and sanitation facilities. The access to improved water and toilet facilities was enhanced by 5.6% and 2.2%, respectively (Table 1).

### 3.1. Overall Equity by Wealth Quintiles

**Figure 1:**
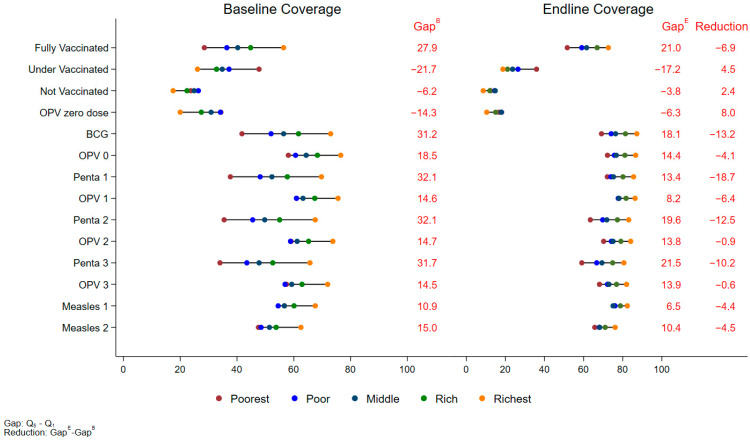
Equity Plot by Wealth Quintiles (Overall).

The overall proportion of fully vaccinated children increased by 20.6% (95% CI: 19.3%, 22.0%) from the baseline coverage of 41.6% (40.6%, 42.7%) to 62.3% (61.2%, 63.3%) at the endline. The proportion of fully vaccinated children increased significantly across all wealth quintiles. In the poorest quintile, the proportion of fully vaccinated children increased from 28.5% (95% CI: 26.7%, 30.3%) at baseline to 51.6% (95% CI: 49.5%, 53.8%) at the endline, an increase of 23.1% (95% CI: 20.5%, 25.8%, *p* < 0.001). In contrast, the richest quintile saw a smaller but still significant increase, from 56.4% (95% CI: 54.6%, 58.2%) to 72.7% (95% CI: 71.1%, 74.2%), an increase of 16.2% (95% CI: 14.0%, 18.4%, *p* < 0.001) (Table 2). The overall vaccination coverage gap between the richest and poorest quintiles narrowed by 6.9%, from 27.9% at the baseline to 21.0% at the endline. A comparable enhancement trend was noted in the coverage of all antigens, leading to decreased equity gaps across the board. For instance, BCG coverage in the poorest quintile rose by 27.4% (from 41.8% at the baseline to 69.2% at the endline). Coverage of Penta3 rose by 25.1% in the poorest quintiles (from 34.0% at the baseline to 59.1% at the endline). This narrowing of the coverage gap between wealth groups indicates a significant reduction in disparities in childhood vaccination coverage (Figure 1).

### 3.2. Regional Equity by Wealth Quintiles

**Figure 2:**
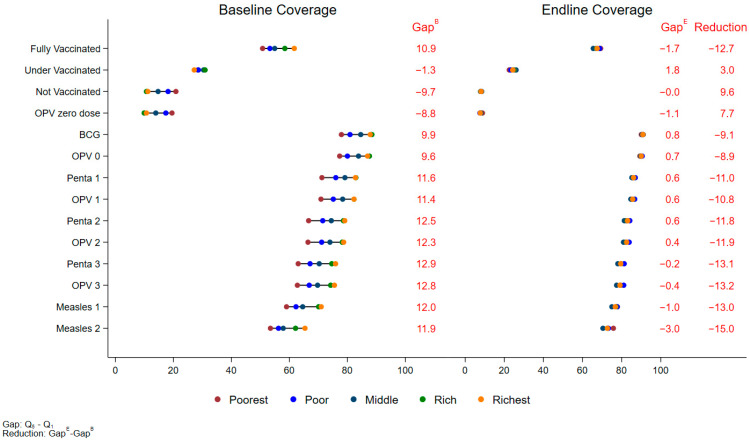
Equity Plot by Wealth Quintiles (Karachi).

**Figure 3:**
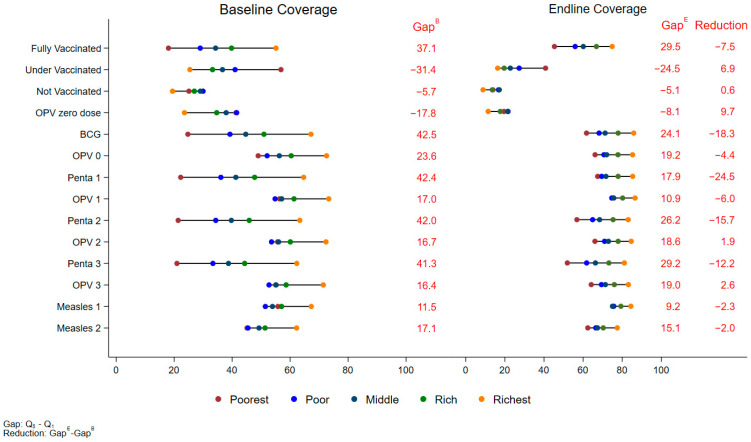
Equity Plot by Wealth Quintiles (KP).

**Figure 4:**
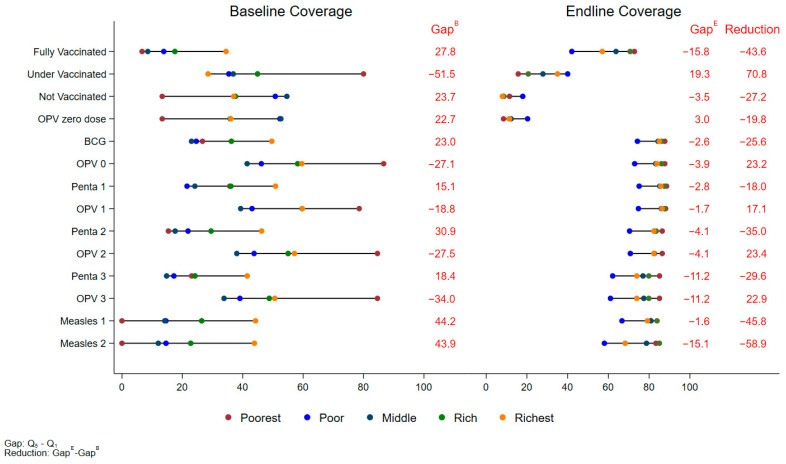
Equity Plot by Wealth Quintiles (Baluchistan).

Regionally, wealth-based disparities in fully vaccinated children were reduced in all areas. In Karachi, the poorest quintile saw an increase in fully vaccinated children from 50.7% (95% CI: 48.5%, 53.0%) at baseline to 69.3% (95% CI: 66.8%, 71.7%) at the endline, an increase of 18.5% (95% CI: 15.3%, 21.8%, *p* < 0.001). The richest quintile in Karachi showed a smaller increase of 5.9% (95% CI: 3.1%, 8.7%, *p* < 0.001), rising from 61.7% (95% CI: 59.7%, 63.6%) to 67.5% (95% CI: 65.4%, 69.6%) at the endline (Table S1). This higher increase in the poorest wealth quintiles has narrowed the gap between the richest and poorest quintiles by 12.7% in Karachi (Figure 2). In KP, the poorest quintile saw a larger increase in fully vaccinated children, from 18.0% (95% CI: 15.9%, 20.1%) to 45.3% (95% CI: 42.6%, 48.1%), a rise of 27.3% (95% CI: 24.0%, 30.6%, *p* < 0.001). The richest quintile in KP saw a 12.1% increase (95% CI: 16.9%, 22.6%, *p* < 0.001) from 55.1% (95% CI: 52.7%, 57.5%) to 74.9% (95% CI: 72.9%, 76.9%) (Table S1). The wealth gap in KP was narrowed by 7.5% (Figure 3). In Baluchistan, the results reflected that the richest quintile showed a 24.6% increase (95% CI: 8.5%, 36.6%, *p* = 0.006), while the poorest quintile saw an increase of 66.1% (95% CI: 53.9%, 78.3%, *p* < 0.001) (Table S1) and the wealth gap narrowed by 27.2% (Figure 4).

### 3.3. Prevalence of Fully Vaccinated Children Across Equity Parameters

Interestingly, the proportion of fully vaccinated children increased regardless of maternal education level. It increased significantly among both literate and illiterate mothers. Among children of literate mothers, the proportion of fully vaccinated children increased from 50.6% (95% CI: 49.2%, 52.0%) to 69.3% (95% CI: 68.0%, 70.5%), an increase of 18.7% (95% CI: 17.0%, 20.3%, *p* < 0.001). Among illiterate mothers, the proportion increased from 34.5% (95% CI: 33.3%, 35.6%) to 55.0% (95% CI: 53.7%, 56.3%), an increase of 20.5% (95% CI: 19.0%, 22.1%, *p* < 0.001) (Table 2).

Moreover, the proportion of fully vaccinated children among males and females increased from the baseline to the endline. Among male children, the proportion of fully vaccinated children increased from 41.7% (95% CI: 40.5%, 42.8%) at the baseline to 63.2% (95% CI: 62.1%, 64.3%) at the endline, an increase of 21.5% (95% CI: 20.1%, 22.9%, *p* < 0.001). Among female children, the proportion increased from 41.6% (95% CI: 40.5%, 42.7%) to 61.1% (95% CI: 60.0%, 62.2%), an increase of 19.5% (95% CI: 18.1%, 20.9%, *p* < 0.001). Among children aged 0–23 months, the proportion of fully vaccinated children increased from 40.1% (95% CI: 39.0%, 41.2%) to 63.5% (95% CI: 62.4%, 64.6%), an increase of 23.4% (95% CI: 22.0%, 24.8%, *p* < 0.001). Among children aged 24–59 months, the proportion increased from 42.5% (95% CI: 41.4%, 43.7%) to 61.5% (95% CI: 60.3%, 62.7%), an increase of 19.0% (95% CI: 17.5%, 20.5%, *p* < 0.001).

The intervention had a greater impact on children from more prominent families. Among children from families with more than six members, the proportion of fully vaccinated children increased from 36.4% (95% CI: 35.2%, 37.6%) to 59.3% (95% CI: 58.0%, 60.7%), an increase of 23.0% (95% CI: 21.3%, 24.6%, *p* < 0.001). Among children from families with six or fewer members, the proportion increased from 50.8% (95% CI: 49.5%, 52.0%) to 66.9% (95% CI: 65.7%, 68.0%), an increase of 16.1% (95% CI: 14.5%, 17.6%, *p* < 0.001) (Table 2).

### 3.4. Prevalence of Under-Vaccinated Children Across Equity Parameters

An improvement of 10.2% (95% CI: −11.4%, −9.1%) was observed in the proportion of under-vaccinated children, with the prevalence decreasing from 35.5% (34.5%, 36.5%) at the baseline to 25.2% (24.4%, 26.1%) at the endline (*p* < 0.001). The poorest quintile saw the highest reduction of 11.8% (95% CI: −14.6%, −9.1%) in the proportion of under-vaccinated children from 47.8% (45.6%, 50.0%) to 35.9% (33.8%, 38.0%) (Table 3). The reduction in under-vaccinated children was observed across all wealth quintiles. The poorest quintile saw a reduction of 11.8% (from 47.8%, 95% CI: 45.6%, 50.0% at the baseline to 35.9%, 95% CI: 33.8%, 38.0% at the endline, *p* < 0.001), while the richest quintile saw a smaller reduction of 7.4% (from 26.1%, 95% CI: 24.7%, 27.5% to 18.7%, 95% CI: 17.5%, 19.9%, *p* < 0.001). The coverage gap between the poorest and richest quintiles decreased, indicating progress in addressing wealth-based disparities in vaccination coverage (Table 3).

Regionally, the gap between the poorest and richest quintiles for under-vaccinated children varied. In Karachi, the poorest quintile saw a reduction of 5.9% (from 28.4%, 95% CI: 27.0%, 29.9% to 22.5%, 95% CI: 20.9%, 24.1%, *p* < 0.001), while the richest quintile saw a reduction of 2.9% (from 27.2%, 95% CI: 25.8%, 28.6% to 24.3%, 95% CI: 22.7%, 25.8%, *p* = 0.0057), as shown in Table S2. In KP, the poorest quintile saw a larger reduction of 16.1% (from 56.9%, 95% CI: 53.9%, 59.8% to 40.8%, 95% CI: 38.1%, 43.5%, *p* < 0.001), while the richest quintile saw a 9.1% reduction (from 25.4%, 95% CI: 23.5%, 27.4% to 16.3%, 95% CI: 14.8%, 17.8%, *p* < 0.001). In Baluchistan, the poorest quintile saw the largest reduction of 64.3% (from 80.0%, 95% CI: 39.0%, 121.0% to 15.7%, 95% CI: 10.8%, 20.7%, *p* = 0.0050) (Table S2).

Children of both literate and illiterate mothers experienced significant reductions in under-vaccinated prevalence. Children of literate mothers saw a reduction of 9.5% (from 31.7%, 95% CI: 30.5%, 32.9% to 22.3%, 95% CI: 21.2%, 23.3%, *p* < 0.001). Children of illiterate mothers experienced a slightly larger reduction of 10.1% (from 38.5%, 95% CI: 37.2%, 39.7% to 28.3%, 95% CI: 27.3%, 29.4%, *p* < 0.001), as shown in Table 3.

Both male and female children saw significant reductions in under-vaccinated prevalence. Male children saw a reduction of 10.5% (from 35.5%, 95% CI: 34.4%, 36.6% to 24.9%, 95% CI: 24.0%, 25.8%, *p* < 0.001). Female children experienced a slightly smaller reduction of 9.8% (from 35.5%, 95% CI: 34.4%, 36.5% to 25.6%, 95% CI: 24.7%, 26.5%, *p* < 0.001). Both age groups saw significant reductions in under-vaccinated prevalence. Children aged 0–23 months saw a reduction of 10.0% (from 36.0%, 95% CI: 35.0%, 37.0% to 26.0%, 95% CI: 25.2%, 26.9%, *p* < 0.001). Children aged 24–59 months saw a reduction of 10.4% (from 35.1%, 95% CI: 34.0%, 36.3% to 24.8%, 95% CI: 23.8%, 25.8%, *p* < 0.001), as shown in Table 3.

Families with six or fewer members saw a reduction of 9.0% in under-vaccinated children (from 31.8%, 95% CI: 30.7%, 33.0% to 22.8%, 95% CI: 21.9%, 23.8%, *p* < 0.001). In contrast, families with more than six members saw a slightly larger reduction of 10.8% (from 37.6%, 95% CI: 36.3%, 38.8% to 26.8%, 95% CI: 25.7%, 27.9%, *p* < 0.001), as shown in Table 3.

### 3.5. Prevalence of Unvaccinated Children Across Equity Parameters

A remarkable overall decrease in unvaccinated children has been observed. At the baseline, 22.9% (95% CI: 21.9%, 23.8%) of children were unvaccinated, which decreased to 12.5% (95% CI: 11.7%, 13.2%) at the endline, representing a 10.4% reduction (95% CI: −11.5%, −9.3%, *p* < 0.001). The largest reduction was seen in the poorest quintile, with an 11.3% decrease (95% CI: −13.6%, −9.1%) in unvaccinated coverage (from 23.7% to 12.4%) (Table 4). By contrast, a reduction of 8.9% (95% CI: −10.6%, −7.1%) was seen in the richest quintile. This reduction in wealth-based disparities is also reflected in narrowing the gap between the richest and poorest quintiles by 2.4% (from 6.2% at the baseline to 3.8% at the endline), as shown in Figure 1.

In the regions, Karachi reported a 6.9% (95% CI: −6.9% (−8.0%, −5.8%) reduction in unvaccinated coverage, whereas for KP and Baluchistan, the higher reduction was reported at 12.0% (95% CI: −13.4%, −10.6%) and 28.1% (95% CI: −45.3%, −10.9%), respectively. A higher reduction in the proportion of unvaccinated children from the poorest quintile has narrowed the gap between the highest and lowest quintiles. A reduction in the gap of 9.1% has been reported for Karachi. Similarly, a 0.6% and 27.2% reduction in the coverage gap between the poorest and richest quintiles for the prevalence of unvaccinated children have been reported for the KP and Baluchistan regions, respectively (Table S3).

Both male and female children saw a reduction in unvaccinated children. Among males, the prevalence decreased by 11.0% (95% CI: −12.1%, −9.8%), while among females, it dropped by 9.7% (95% CI: −10.8%, −8.5%). The most substantial reductions were seen in younger children (0–23 months), where zero-dose prevalence decreased by 13.4% (95% CI: −14.5%, −12.3%). Among older children (24–59 months), the decrease was 8.6% (95% CI: −9.8%, −7.4%) (Table 4).

The reduction in unvaccinated children was observed among both children of literate and illiterate mothers. Children of literate mothers saw a reduction of 9.2% (from 17.7%, 95% CI: 16.6%, 18.8% to 8.5%, 95% CI: 7.7%, 9.2%, *p* < 0.001). Children of illiterate mothers experienced a similar reduction of 10.4% (from 27.1%, 95% CI: 25.9%, 28.2% to 16.7%, 95% CI: 15.6%, 17.7%, *p* < 0.001), as shown in Table 4.

Family size showed varying impacts on vaccination coverage. Families with six or fewer members saw a reduction of 7.1% (from 17.4%, 95% CI: 16.4%, 18.4% to 10.3%, 95% CI: 9.6%, 11.0%, *p* < 0.001) in unvaccinated children, while families with more than six members saw a larger reduction of 12.2% (from 26.1%, 95% CI: 24.9%, 27.2% to 13.9%, 95% CI: 13.0%, 14.8%, *p* < 0.001) (Table 4).

### 3.6. Prevalence of Zero-Dose Children Across Equity Parameters

A significant reduction in polio zero-dose children was achieved, with prevalence falling by 13.5% (95% CI: −14.8%, −12.2%), from 29.2% (95% CI: 28.0%, 30.3%) at the baseline to 15.6% (14.8%, 16.5%) at the endline. The largest reductions were seen in the poorest quintile, which depicts a 17.6% (95% CI: −20.5%, −14.7%) reduction in polio zero-dose prevalence. In comparison, the richest quintile saw a 9.6% reduction (95% CI: −11.5%, −7.7%) (Table 5). This narrowed the gap between the poorest and richest quintiles by 8.0%, reducing from 14.3% at the baseline to 6.3% at the endline (Figure 1).

The impact on polio zero-dose prevalence across the wealth quintiles regionally varied. In Karachi, the poorest quintile saw a reduction of 10.7% (from 19.5%, 95% CI: 17.5%, 21.5% to 8.8%, 95% CI: 7.1%, 10.6%, *p* < 0.001), while the richest quintile saw a smaller reduction of 3.0% (from 10.7%, 95% CI: 9.3%, 12.0% to 7.7%, 95% CI: 6.5%, 8.9%, *p* = 0.0011), as shown in Table S4. In KP, the poorest quintile experienced a significant reduction of 21.8% (from 41.3%, 95% CI: 37.9%, 44.8% to 19.5%, 95% CI: 17.6%, 21.5%, *p* < 0.001), while the richest quintile saw a reduction of 12.1% (from 23.6%, 95% CI: 21.4%, 25.7% to 11.4%, 95% CI: 10.0%, 12.8%, *p* < 0.001). In Baluchistan, the poorest quintile saw a non-significant reduction of 4.8% (from 13.3%, 95% CI: −14.0%, 40.6% to 8.5%, 95% CI: 4.6%, 12.4%, *p* = 0.717), while the richest quintile saw a significant reduction of 24.6% (from 36.1%, 95% CI: 19.8%, 52.3% to 11.5%, 95% CI: 1.9%, 21.0%, *p* = 0.021).

Children of both literate and illiterate mothers saw significant reductions in polio zero-dose prevalence. Children of literate mothers saw a reduction of 13.0% (from 24.5%, 95% CI: 23.2%, 25.9% to 11.6%, 95% CI: 10.7%, 12.4%, *p* < 0.001), while children of illiterate mothers experienced the same magnitude of reduction at 13.0% (from 32.9%, 95% CI: 31.5%, 34.2% to 19.8%, 95% CI: 18.7%, 21.0%, *p* < 0.001), as shown in Table 5.

Both male and female children showed significant reductions in the prevalence of polio zero-dose children. Male children saw a reduction of 14.5% (from 29.7%, 95% CI: 28.5%, 30.9% to 15.2%, 95% CI: 14.3%, 16.0%, *p* < 0.001). Female children experienced a slightly smaller reduction of 12.3% (from 28.5%, 95% CI: 27.4%, 29.6% to 16.2%, 95% CI: 15.3%, 17.1%, *p* < 0.001). Both age groups saw significant reductions in polio zero-dose prevalence. Children aged 0–23 months experienced the largest reduction, with a 16.1% decrease (from 26.3%, 95% CI: 25.2%, 27.4% to 10.2%, 95% CI: 9.5%, 10.9%, *p* < 0.001). Children aged 24–59 months experienced a smaller reduction of 11.9% (from 30.8%, 95% CI: 29.6%, 32.0% to 18.9%, 95% CI: 17.9%, 20.0%, *p* < 0.001), as shown in Table 5.

Both small and large families saw significant reductions in polio zero-dose prevalence. Families with six or fewer members saw a reduction of 6.8% (from 19.5%, 95% CI: 18.4%, 20.5% to 12.7%, 95% CI: 11.8%, 13.5%, *p* < 0.001), while families with more than six members saw a more significant reduction of 17.2% (from 34.7%, 95% CI: 33.4%, 36.1% to 17.5%, 95% CI: 16.5%, 18.5%, *p* < 0.001), as shown in Table 5.

**Figure 5:**
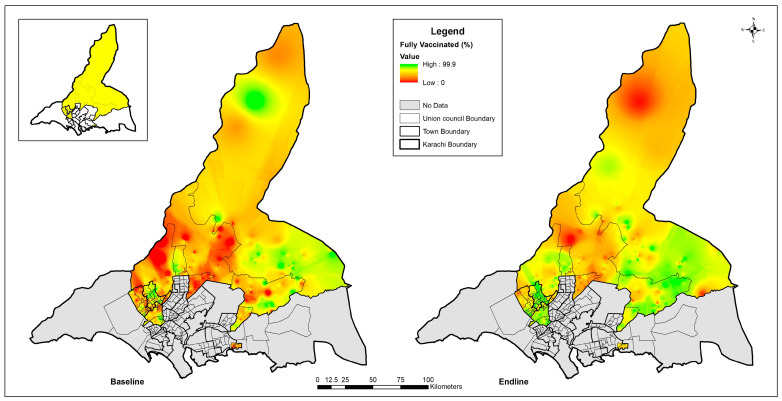
Cluster-level geospatial mapping of fully vaccinated children.

**Figure 6:**
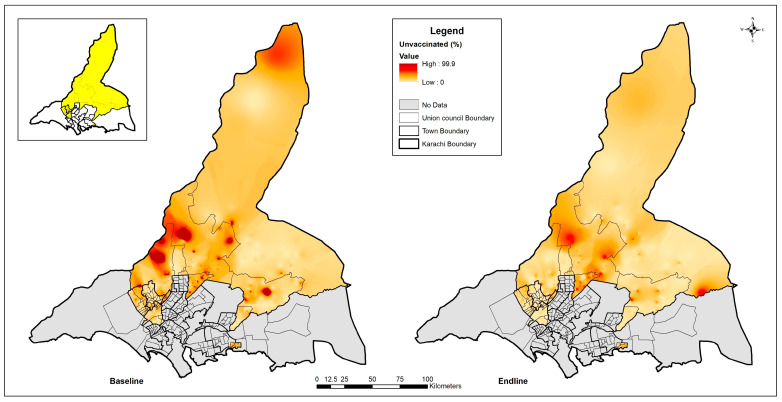
Cluster-level geospatial mapping of unvaccinated children.

Furthermore, utilizing the cluster-level geospatial data, we identified areas with significant clustering of fully vaccinated children (Figure 5) and unvaccinated children (Figure 6). Additionally, spatial autocorrelation analysis using Moran’s I was conducted for the baseline and endline datasets of unvaccinated and fully vaccinated children. At the cluster level, Moran’s I for unvaccinated individuals decreased from 0.148 (z = 45.26, *p* < 0.05) to 0.053 (z = 21.47, *p* < 0.05) at the endline. Similarly, for fully vaccinated individuals, Moran’s I decreased from 0.171 (z = 52.16, *p* < 0.05) at the baseline to 0.101 (z = 40.41, *p* < 0.05) at the endline. These results indicate statistically significant spatial clustering at both time points, with a reduction in clustering over time, suggesting that the targeted interventions effectively address spatial disparities in vaccination coverage.

## 4. Discussion

The results of this study show substantial improvements in immunization coverage among children from various demographic and socio-economic groups. The children from the lowest wealth quintiles have benefited the most.

Integrating community engagement, health camps, and outreach to marginalized populations has led to improved immunization coverage, reductions in zero-dose children, and a narrowing of vaccination coverage and healthcare-access inequities. This reduction in wealth inequity is critical, as socioeconomic status has consistently been shown to be one of the strongest determinants of immunization coverage [5].

Our results show that the most considerable reductions in inequity were in the poorest wealth quintiles, with a reduction of up to 17.6% in polio zero-dose children and a substantial increase in BCG, Penta 3, and Measles 1 coverage. The reduction across different wealth quintiles in immunization coverage is consistent with previous studies, which have shown that disparities in immunization coverage and health services can be reduced by employing targeted interventions focusing on underserved communities [5]. The existing literature indicates that financial constraints, geographic isolation, and lack of awareness make children from lower socio-economic backgrounds less likely to access routine immunization services [14,15]. The gender disparity in vaccination coverage was also reduced. Both male and female children had a reduction in zero-dose prevalence, although the reduction was slightly more pronounced for male children. This finding is consistent with other studies from similar settings, which have found minimal but persistent gender differences in vaccination coverage [16].

The results indicate that urban areas like Karachi have higher vaccination rates due to better health infrastructure. However, we achieved equitable immunization access through targeted interventions in conflict-affected regions. In contrast to Karachi, Khyber Pakhtunkhwa (KPK) and Baluchistan showed larger reductions in zero-dose children, highlighting the greater number of unvaccinated individuals in these areas. This suggests that while Karachi’s campaigns refined coverage, the interventions in KPK and Baluchistan had a more substantial impact on under-immunized populations, where access to primary healthcare is more challenging [17]. Although we observed a reduction in the equity gap through our approach, disparities in vaccination coverage are still prevalent [18]. The success of the study and an overall improvement in vaccination rates and equity can be attributed to the holistic nature of the project. Combining access to healthcare facilities, building trust through effective community engagement, and mobilizing through the inclusion of community leaders and representatives as focal persons, as well as increasing awareness about vaccination, addressed the most prevalent barriers to vaccine uptake. The current analysis also provides the foundation for further work on equity parameters while acting as a baseline measurement.

Several previous studies have stressed the urgent need to address the healthcare disparities and the immense importance of prioritizing the efforts to reach the poorest and most underserved children [19,20,21]. Focusing on these groups is not only practical but also cost-efficient. Our findings allow us to bridge these gaps by implementing vaccination programs integrated with comprehensive maternal and child healthcare services.

To improve vaccine coverage and reduce disease-burden disparities among the poorest populations, we should focus on targeted efforts to increase access and enhance the performance of immunization programs while also minimizing out-of-pocket expenses. Evidence from national and global initiatives, such as those led by Gavi, supports this approach [22,23]. These approaches are in consensus with existing research and support the WHO’s Global Routine Immunization Strategies and Practices (GRISP) [23], which emphasizes delivering immunization through fixed health facilities and mobile outreach services. This framework encompasses an inclusive model of locating and reaching underserved populations, enhancing service availability, and fostering vaccination demand through public health campaigns.

The key limitation of this analysis is that the coverage estimates were mostly dependent on family statements and dosages delivered at the site. This dependence on family reporting may bring biases into coverage estimates, as it is frequently the primary source of data in conflict-affected insecure regions. Future techniques may also rely on immunization records, whether paper or electronic. Moving forward, a key priority is to establish long-term coordination and resourcing with local institutions, as they are uniquely equipped to develop practical solutions for their communities.

## 5. Conclusions

This study highlights the essential role of equitable healthcare interventions in improving immunization rates. To reduce the equity gap in Pakistan, outreach activities integrating vaccination with maternal and child health services should be expanded in underserved areas. Strengthening community engagement through partnerships with community influencers can address vaccine hesitancy and promote uptake, especially in rural and marginalized communities. Leveraging digital tools like geospatial mapping to target low-performing pockets for immunization and incentivizing health workers can ensure sustainable and equitable access to routine immunization services.

## Figures and Tables

**Table 1:** Socio-demographic characteristics of children under 5 and their families at baseline and endline.

Characteristics	BL % (95% CI)	EL % (95% CI)	EL-BL % (95% CI)
**Gender**			
Male	54.9% (54.6%, 55.2%)	55.9% (55.6%, 56.2%)	1.0% (0.6%, 1.4%)
Female	45.1% (44.8%, 45.4%)	44.1% (43.8%, 44.4%)	−1.0% (−1.4%, −0.6%)
**Age (months)**			
0–23	36.6% (36.3%, 37.0%)	37.8% (37.3%, 38.3%)	1.2% (0.6%, 1.8%)
24–59	63.4% (63.0%, 63.7%)	62.2% (61.7%, 62.7%)	−1.2% (−1.8%, −0.6%)
**Wealth quintile**			
Poorest	19.0% (18.1%, 19.9%)	20.5% (19.7%, 21.4%)	1.5% (0.5%, 2.5%)
Poor	19.4% (18.8%, 20.0%)	20.1% (19.5%, 20.7%)	0.7% (−0.1%, 1.5%)
Middle	19.9% (19.3%, 20.4%)	19.9% (19.3%, 20.5%)	0.1% (−0.7%, 0.8%)
Rich	20.3% (19.7%, 21.0%)	19.6% (19.0%, 20.2%)	−0.7% (−1.5%, 0.1%)
Richest	21.4% (20.4%, 22.3%)	19.8% (19.0%, 20.6%)	−1.5% (−2.6%, −0.5%)
**Mother education**			
Literate	44.5% (43.6%, 45.5%)	50.8% (49.8%, 51.8%)	6.3% (5.1%, 7.5%)
Illiterate	55.5% (54.5%, 56.4%)	49.2% (48.2%, 50.2%)	−6.3% (−7.5%, −5.1%)
**Family Size**			
≤6	36.6% (35.8%, 37.4%)	38.9% (38.0%, 39.7%)	2.3% (1.3%, 3.2%)
>6	63.4% (62.6%, 64.2%)	61.1% (60.3%, 62.0%)	−2.3% (−3.2%, −1.3%)
**Improved water**	80.7% (79.7%, 81.8%)	86.4% (85.5%, 87.2%)	5.6% (4.5%, 6.8%)
**Improved toilet facility**	65.1% (63.9%, 66.4%)	67.3% (66.2%, 68.5%)	2.2% (0.8%, 3.6%)

BL, Baseline; EL, Endline; CI, Confidence Interval.

**Table 2:** Prevalence of fully vaccinated children and changes across equity parameters.

Equity Parameters	BL % (95% CI)	EL % (95% CI)	EL-BL % (95% CI)	*p*-Values
**Overall**	41.6% (40.6%, 42.7%)	62.3% (61.2%, 63.3%)	20.6% (19.3%, 22.0%)	<0.001
**Gender**				
Male	41.7% (40.5%, 42.8%)	63.2% (62.1%, 64.3%)	21.5% (20.1%, 22.9%)	<0.001
Female	41.6% (40.5%, 42.7%)	61.1% (60.0%, 62.2%)	19.5% (18.1%, 20.9%)	<0.001
**Age (months)**				
0–23	40.1% (39.0%, 41.2%)	63.5% (62.4%, 64.6%)	23.4% (22.0%, 24.8%)	<0.001
24–59	42.5% (41.4%, 43.7%)	61.5% (60.3%, 62.7%)	19.0% (17.5%, 20.5%)	<0.001
**Wealth quintile**				
Poorest	28.5% (26.7%, 30.3%)	51.6% (49.5%, 53.8%)	23.1% (20.5%, 25.8%)	<0.001
Poor	36.4% (34.8%, 38.0%)	59.1% (57.4%, 60.8%)	22.7% (20.4%, 24.9%)	<0.001
Middle	40.3% (38.8%, 41.8%)	61.5% (59.9%, 63.1%)	21.3% (19.2%, 23.3%)	<0.001
Rich	44.8% (43.2%, 46.4%)	66.9% (65.4%, 68.4%)	22.1% (20.1%, 24.2%)	<0.001
Richest	56.4% (54.6%, 58.2%)	72.7% (71.1%, 74.2%)	16.2% (14.0%, 18.4%)	<0.001
**Mother education**				
Literate	50.6% (49.2%, 52.0%)	69.3% (68.0%, 70.5%)	18.7% (17.0%, 20.3%)	<0.001
Illiterate	34.5% (33.3%, 35.6%)	55.0% (53.7%, 56.3%)	20.5% (19.0%, 22.1%)	<0.001
**Family Size**				
≤6	50.8% (49.5%, 52.0%)	66.9% (65.7%, 68.0%)	16.1% (14.5%, 17.6%)	<0.001
>6	36.4% (35.2%, 37.6%)	59.3% (58.0%, 60.7%)	23.0% (21.3%, 24.6%)	<0.001

BL, Baseline; EL, Endline; CI, Confidence Interval.

**Table 3:** Prevalence of under-vaccinated children and changes across equity.

Equity Parameters	BL % (95% CI)	EL % (95% CI)	EL-BL % (95% CI)	*p*-Values
**Overall**	35.5% (34.5%, 36.5%)	25.2% (24.4%, 26.1%)	−10.2% (−11.4%, −9.1%)	<0.001
**Gender**				
Male	35.5% (34.4%, 36.6%)	24.9% (24.0%, 25.8%)	−10.5% (−11.8%, −9.3%)	<0.001
Female	35.5% (34.4%, 36.5%)	25.6% (24.7%, 26.5%)	−9.8% (−11.0%, −8.6%)	<0.001
**Age (months)**				
0–23	36.0% (35.0%, 37.0%)	26.0% (25.2%, 26.9%)	−10.0% (−11.2%, −8.8%)	<0.001
24–59	35.1% (34.0%, 36.3%)	24.8% (23.8%, 25.8%)	−10.4% (−11.7%, −9.0%)	<0.001
**Wealth quintile**				
Poorest	47.8% (45.6%, 50.0%)	35.9% (33.8%, 38.0%)	−11.8% (−14.6%, −9.1%)	<0.001
Poor	37.2% (35.6%, 38.8%)	26.4% (25.0%, 27.8%)	−10.8% (−12.7%, −8.8%)	<0.001
Middle	34.8% (33.3%, 36.3%)	23.7% (22.5%, 24.9%)	−11.1% (−13.0%, −9.3%)	<0.001
Rich	32.8% (31.4%, 34.2%)	21.1% (20.0%, 22.1%)	−11.7% (−13.5%, −10.0%)	<0.001
Richest	26.1% (24.7%, 27.5%)	18.7% (17.5%, 19.9%)	−7.4% (−9.2%, −5.6%)	<0.001
**Mother education**				
Literate	31.7% (30.5%, 32.9%)	22.3% (21.2%, 23.3%)	−9.5% (−10.9%, −8.0%)	<0.001
Illiterate	38.5% (37.2%, 39.7%)	28.3% (27.3%, 29.4%)	−10.1% (−11.6%, −8.7%)	<0.001
**Family Size**				
≤6	31.8% (30.7%, 33.0%)	22.8% (21.9%, 23.8%)	−9.0% (−10.3%, −7.6%)	<0.001
>6	37.6% (36.3%, 38.8%)	26.8% (25.7%, 27.9%)	−10.8% (−12.3%, −9.3%)	<0.001

BL, Baseline; EL, Endline; CI, Confidence Interval.

**Table 4:** Prevalence of unvaccinated children and changes across equity parameters.

Equity Parameters	BL % (95% CI)	EL % (95% CI)	EL-BL % (95% CI)	*p*-Values
**Overall**	22.9% (21.9%, 23.8%)	12.5% (11.7%, 13.2%)	−10.4% (−11.5%, −9.3%)	<0.001
**Gender**				
Male	22.9% (21.9%, 23.9%)	11.9% (11.1%, 12.6%)	−11.0% (−12.1%, −9.8%)	<0.001
Female	22.9% (22.0%, 23.9%)	13.3% (12.5%, 14.1%)	−9.7% (−10.8%, −8.5%)	<0.001
**Age**				
0–23	23.9% (22.9%, 24.9%)	10.5% (9.8%, 11.1%)	−13.4% (−14.5%, −12.3%)	<0.001
24–59	22.3% (21.3%, 23.3%)	13.7% (12.9%, 14.6%)	−8.6% (−9.8%, −7.4%)	<0.001
**Wealth quintile**				
Poorest	23.7% (21.9%, 25.6%)	12.4% (11.1%, 13.8%)	−11.3% (−13.6%, −9.1%)	<0.001
Poor	26.4% (24.8%, 27.9%)	14.5% (13.3%, 15.7%)	−11.9% (−13.8%, −10.0%)	<0.001
Middle	24.9% (23.5%, 26.4%)	14.8% (13.6%, 16.0%)	−10.1% (−11.9%, −8.4%)	<0.001
Rich	22.4% (21.0%, 23.8%)	12.0% (10.9%, 13.1%)	−10.4% (−12.0%, −8.7%)	<0.001
Richest	17.5% (15.9%, 19.1%)	8.6% (7.7%, 9.6%)	−8.9% (−10.6%, −7.1%)	<0.001
**Mother education**				
Literate	17.7% (16.6%, 18.8%)	8.5% (7.7%, 9.2%)	−9.2% (−10.5%, −8.0%)	<0.001
Illiterate	27.1% (25.9%, 28.2%)	16.7% (15.6%, 17.7%)	−10.4% (−11.8%, −9.0%)	<0.001
**Family Size**				
≤6	17.4% (16.4%, 18.4%)	10.3% (9.6%, 11.0%)	−7.1% (−8.2%, −6.0%)	<0.001
>6	26.1% (24.9%, 27.2%)	13.9% (13.0%, 14.8%)	−12.2% (−13.5%, −10.8%)	<0.001

BL, Baseline; EL, Endline; CI, Confidence Interval.

**Table 5:** Prevalence of polio zero-dose children and changes across equity parameters.

Equity Parameters	BL % (95% CI)	EL % (95% CI)	EL-BL % (95% CI)	*p*-Values
**Overall**	29.2% (28.0%, 30.3%)	15.6% (14.8%, 16.5%)	−13.5% (−14.8%, −12.2%)	<0.001
**Gender**				
Male	29.7% (28.5%, 30.9%)	15.2% (14.3%, 16.0%)	−14.5% (−15.9%, −13.1%)	<0.001
Female	28.5% (27.4%, 29.6%)	16.2% (15.3%, 17.1%)	−12.3% (−13.6%, −10.9%)	<0.001
**Age**				
0–23	26.3% (25.2%, 27.4%)	10.2% (9.5%, 10.9%)	−16.1% (−17.3%, −14.9%)	<0.001
24–59	30.8% (29.6%, 32.0%)	18.9% (17.9%, 20.0%)	−11.9% (−13.3%, −10.4%)	<0.001
**Wealth quintile**				
Poorest	34.3% (31.8%, 36.8%)	16.7% (15.2%, 18.2%)	−17.6% (−20.5%, −14.7%)	<0.001
Poor	34.2% (32.3%, 36.0%)	18.0% (16.6%, 19.4%)	−16.2% (−18.4%, −13.9%)	<0.001
Middle	30.9% (29.2%, 32.5%)	17.9% (16.6%, 19.3%)	−13.0% (−15.0%, −11.0%)	<0.001
Rich	27.4% (25.8%, 29.0%)	15.0% (13.8%, 16.2%)	−12.4% (−14.2%, −10.6%)	<0.001
Richest	20.0% (18.4%, 21.7%)	10.4% (9.4%, 11.5%)	−9.6% (−11.5%, −7.7%)	<0.001
**Mother education**				
Literate	24.5% (23.2%, 25.9%)	11.6% (10.7%, 12.4%)	−13.0% (−14.5%, −11.4%)	<0.001
Illiterate	32.9% (31.5%, 34.2%)	19.8% (18.7%, 21.0%)	−13.0% (−14.6%, −11.4%)	<0.001
**Family Size**				
≤6	19.5% (18.4%, 20.5%)	12.7% (11.8%, 13.5%)	−6.8% (−8.0%, −5.5%)	<0.001
>6	34.7% (33.4%, 36.1%)	17.5% (16.5%, 18.5%)	−17.2% (−18.9%, −15.6%)	<0.001

BL, Baseline; EL, Endline; CI, Confidence Interval.

## Data Availability

The data presented in this study are available on request from the corresponding author. The data are not publicly available due to privacy and ethical concerns.

## Supplementary Materials

The following supporting information can be downloaded at: https://www.mdpi.com/article/10.3390/vaccines12121340/s1. Table S1: Fully vaccinated prevalence and change across equity parameters by region; Table S2: Under-vaccinated, prevalence, and change across equity parameters by region; Table S3: Not immunized prevalence and change across equity parameters by region; Table S4: Polio zero-dose prevalence and change across equity parameters by region.
