# Supplementary material for: Bridging the Vaccination Equity Gap: A Community-Driven Approach to Reduce Vaccine Inequities in Polio High-Risk Areas of Pakistan
Source: Vaccines (Basel). 2024 Nov 28;12(12):1340. doi: 10.3390/vaccines12121340 (PMC11680313; doi:10.3390/vaccines12121340)
Supplement: Supplementary file 1 [file vaccines-12-01340-s001.zip › vaccines-3309482-supplementary.pdf]

Table S1: Fully Vaccinated prevalence and change across equity parameters by region

Equity Parameters	Karachi				KP				Baluchistan			
	BL % (95 % CI)	EL % (95 % CI)	EL-BL % (95 % CI)	P-Values	BL % (95 % CI)	EL % (95 % CI)	EL-BL % (95 % CI)	P-Values	BL % (95 % CI)	EL % (95 % CI)	EL-BL % (95 % CI)	P-Values
**Overall**	55.9% (54.8%, 57.0%)	67.6% (66.5%, 68.8%)	11.7% (10.2%, 13.3%)	<0.001	35.6% (34.2%, 37.0%)	60.4% (59.0%, 61.8%)	24.8% (23.1%, 26.6%)	<0.001	26.0% (14.2%, 37.8%)	60.4% (50.1%, 70.8%)	34.4% (22.3%, 46.6%)	0.0001
**Wealth Quintiles**												
Poorest	50.7% (48.5%, 53.0%)	69.3% (66.8%, 71.7%)	18.5% (15.3%, 21.8%)	<0.001	18.0% (15.9%, 20.1%)	45.3% (42.6%, 48.1%)	27.3% (24.0%, 30.6%)	<0.001	6.7% (-7.0%, 20.3%)	72.8% (66.8%, 78.8%)	66.1% (53.9%, 78.3%)	<0.001
Poor	53.3% (51.4%, 55.1%)	68.8% (67.0%, 70.6%)	15.5% (13.0%, 18.0%)	<0.001	29.0% (27.0%, 31.0%)	55.9% (53.8%, 58.1%)	26.9% (24.0%, 29.8%)	<0.001	13.8% (3.5%, 24.2%)	42.0% (30.4%, 53.7%)	28.2% (18.3%, 38.1%)	<0.001
Middle	54.9% (53.2%, 56.6%)	65.5% (63.7%, 67.3%)	10.6% (8.2%, 13.0%)	<0.001	34.3% (32.3%, 36.2%)	60.1% (58.0%, 62.1%)	25.8% (23.1%, 28.5%)	<0.001	8.6% (0.8%, 16.3%)	63.7% (54.0%, 73.5%)	55.2% (40.3%, 70.1%)	<0.001
Rich	58.4% (56.8%, 60.1%)	67.0% (65.3%, 68.8%)	8.6% (6.3%, 11.0%)	<0.001	39.8% (37.7%, 41.9%)	66.8% (64.9%, 68.7%)	27.0% (24.4%, 29.6%)	<0.001	17.5% (8.2%, 26.8%)	70.7% (57.6%, 83.8%)	53.1% (36.9%, 69.4%)	<0.001
Richest	61.7% (59.7%, 63.6%)	67.5% (65.4%, 69.6%)	5.9% (3.1%, 8.7%)	<0.001	55.1% (52.7%, 57.5%)	74.9% (72.9%, 76.9%)	19.7% (16.9%, 22.6%)	<0.001	34.5% (21.1%, 47.9%)	57.0% (42.0%, 72.0%)	22.5% (8.5%, 36.6%)	0.006
**Mother Education**												
Literate	68.7% (67.7%, 69.8%)	77.8% (76.8%, 78.7%)	9.0% (7.6%, 10.5%)	<0.001	41.5% (39.7%, 43.4%)	66.5% (64.9%, 68.0%)	24.9% (22.7%, 27.1%)	<0.001	42.6% (15.6%, 69.6%)	79.6% (62.1%, 97.1%)	37.0% (22.9%, 51.1%)	<0.001
Illiterate	43.9% (42.6%, 45.2%)	58.1% (56.6%, 59.6%)	14.2% (12.3%, 16.2%)	<0.001	31.0% (29.4%, 32.5%)	53.7% (52.0%, 55.4%)	22.7% (20.7%, 24.8%)	<0.001	23.5% (13.6%, 33.4%)	59.6% (49.3%, 70.0%)	36.1% (24.7%, 47.6%)	<0.001
**Gender**												
Male	57.2% (56.0%, 58.4%)	68.3% (67.0%, 69.5%)	11.1% (9.4%, 12.8%)	<0.001	35.6% (34.2%, 37.1%)	61.6% (60.1%, 63.0%)	25.9% (24.1%, 27.7%)	<0.001	25.6% (12.6%, 38.7%)	61.2% (50.9%, 71.6%)	35.6% (22.7%, 48.5%)	<0.001
Female	54.6% (53.4%, 55.9%)	67.0% (65.7%, 68.2%)	12.3% (10.6%, 14.1%)	<0.001	35.5% (34.0%, 36.9%)	58.8% (57.3%, 60.3%)	23.3% (21.5%, 25.1%)	<0.001	26.4% (15.6%, 37.1%)	59.5% (48.5%, 70.6%)	33.2% (21.0%, 45.4%)	<0.001
**Age**												
0-23	51.6% (50.4%, 52.8%)	61.7% (60.4%, 63.0%)	10.1% (8.4%, 11.8%)	<0.001	34.4% (33.0%, 35.9%)	64.3% (62.9%, 65.8%)	29.9% (28.1%, 31.7%)	<0.001	15.6% (6.4%, 24.9%)	57.8% (48.4%, 67.2%)	42.2% (30.5%, 53.8%)	<0.001
24-59	59.0% (57.7%, 60.3%)	71.5% (70.3%, 72.8%)	12.6% (10.8%, 14.3%)	<0.001	36.1% (34.6%, 37.6%)	58.1% (56.5%, 59.6%)	21.9% (20.0%, 23.8%)	<0.001	33.3% (19.4%, 47.2%)	65.3% (51.1%, 79.5%)	32.1% (16.1%, 48.1%)	0.001
**Family Size**												
<=6	61.1% (59.8%, 62.3%)	72.0% (70.8%, 73.2%)	11.0% (9.3%, 12.6%)	<0.001	44.6% (42.8%, 46.5%)	63.7% (62.1%, 65.3%)	19.1% (16.8%, 21.3%)	<0.001	25.9% (15.8%, 35.9%)	77.7% (70.7%, 84.7%)	51.8% (39.5%, 64.1%)	<0.001
>6	51.6% (50.3%, 52.9%)	62.2% (60.8%, 63.7%)	10.7% (8.8%, 12.5%)	<0.001	31.1% (29.6%, 32.6%)	58.7% (57.1%, 60.3%)	27.6% (25.6%, 29.6%)	<0.001	26.0% (13.3%, 38.7%)	55.7% (44.8%, 66.5%)	29.6% (17.1%, 42.2%)	0.0005

Table S2: Under vaccinated, prevalence and change across equity parameters by region

Equity Parameters	Karachi				KP				Baluchistan			
	BL % (95 % CI)	EL % (95 % CI)	EL-BL % (95 % CI)	P-Values	BL % (95 % CI)	EL % (95 % CI)	EL-BL % (95 % CI)	P-Values	BL % (95 % CI)	EL % (95 % CI)	EL-BL % (95 % CI)	P-Values
**Overall**	29.0% (28.3%, 29.8%)	24.2% (23.4%, 25.1%)	-4.8% (-5.9%, -3.7%)	<0.001	38.4% (36.9%, 39.8%)	25.6% (24.4%, 26.7%)	-12.8% (-14.4%, -11.2%)	<0.001	35.0% (18.9%, 51.0%)	28.6% (21.2%, 36.1%)	-6.3% (-18.9%, 6.3%)	0.2872
**Wealth Quintiles**												
Poorest	28.4% (27.0%, 29.9%)	22.5% (20.9%, 24.1%)	-5.9% (-8.1%, -3.7%)	<0.001	56.9% (53.9%, 59.8%)	40.8% (38.1%, 43.5%)	-16.1% (-19.8%, -12.4%)	<0.001	80.0% (39.0%, 121.0%)	15.7% (10.8%, 20.7%)	-64.3% (-103.7%, -24.8%)	0.0050
Poor	28.6% (27.3%, 29.9%)	23.3% (22.0%, 24.7%)	-5.3% (-7.1%, -3.4%)	<0.001	41.0% (38.9%, 43.2%)	27.3% (25.5%, 29.1%)	-13.7% (-16.3%, -11.1%)	<0.001	35.4% (1.1%, 69.7%)	40.0% (29.2%, 50.8%)	4.6% (-26.2%, 35.4%)	0.7422
Middle	30.4% (29.0%, 31.7%)	26.0% (24.6%, 27.5%)	-4.3% (-6.3%, -2.4%)	<0.001	36.7% (34.6%, 38.7%)	22.8% (21.2%, 24.3%)	-13.9% (-16.3%, -11.5%)	<0.001	36.8% (16.6%, 57.1%)	27.9% (21.1%, 34.6%)	-9.0% (-26.4%, 8.4%)	0.2730
Rich	30.9% (29.5%, 32.2%)	25.1% (23.7%, 26.4%)	-5.8% (-7.7%, -3.9%)	<0.001	33.2% (31.3%, 35.2%)	19.6% (18.3%, 21.0%)	-13.6% (-15.9%, -11.3%)	<0.001	44.9% (26.6%, 63.2%)	20.7% (11.3%, 30.1%)	-24.2% (-39.2%, -9.2%)	0.0053
Richest	27.2% (25.8%, 28.6%)	24.3% (22.7%, 25.8%)	-2.9% (-4.9%, -0.8%)	0.0057	25.4% (23.5%, 27.4%)	16.3% (14.8%, 17.8%)	-9.1% (-11.5%, -6.8%)	<0.001	28.5% (12.8%, 44.2%)	35.0% (23.3%, 46.7%)	6.5% (-8.2%, 21.2%)	0.3438
**Mother Education**												
Literate	25.7% (24.9%, 26.6%)	19.1% (18.2%, 19.9%)	-6.7% (-7.9%, -5.5%)	<0.001	34.7% (33.0%, 36.4%)	23.3% (22.0%, 24.7%)	-11.4% (-13.4%, -9.4%)	<0.001	31.8% (13.3%, 50.4%)	13.0% (5.5%, 20.4%)	-18.9% (-37.4%, -0.3%)	0.0471
Illiterate	32.2% (31.2%, 33.1%)	29.1% (28.0%, 30.2%)	-3.1% (-4.5%, -1.6%)	<0.001	41.1% (39.5%, 42.8%)	28.0% (26.6%, 29.4%)	-13.2% (-15.1%, -11.2%)	<0.001	35.4% (19.0%, 51.8%)	29.3% (21.9%, 36.7%)	-6.1% (-19.2%, 6.9%)	0.3148
**Gender**												
Male	28.5% (27.7%, 29.3%)	23.9% (23.0%, 24.8%)	-4.6% (-5.9%, -3.4%)	<0.001	38.3% (36.9%, 39.8%)	25.2% (24.1%, 26.4%)	-13.1% (-14.8%, -11.4%)	<0.001	35.0% (18.6%, 51.4%)	28.6% (20.1%, 37.1%)	-6.4% (-20.1%, 7.3%)	0.3179
Female	29.6% (28.8%, 30.5%)	24.6% (23.7%, 25.6%)	-5.0% (-6.2%, -3.7%)	<0.001	38.4% (36.9%, 39.9%)	26.0% (24.8%, 27.2%)	-12.4% (-14.1%, -10.8%)	<0.001	34.9% (18.7%, 51.1%)	28.7% (21.5%, 35.9%)	-6.2% (-18.4%, 5.9%)	0.2766
**Age**												
0-23	32.6% (31.7%, 33.5%)	29.4% (28.4%, 30.4%)	-3.2% (-4.6%, -1.8%)	<0.001	37.7% (36.3%, 39.1%)	24.6% (23.5%, 25.8%)	-13.1% (-14.7%, -11.5%)	<0.001	41.9% (25.6%, 58.2%)	30.2% (24.4%, 36.1%)	-11.7% (-25.0%, 1.6%)	0.0788
24-59	26.5% (25.7%, 27.3%)	20.8% (19.9%, 21.7%)	-5.7% (-6.9%, -4.5%)	<0.001	38.7% (37.1%, 40.2%)	26.1% (24.8%, 27.4%)	-12.6% (-14.4%, -10.8%)	<0.001	30.1% (14.2%, 46.0%)	25.7% (13.4%, 38.0%)	-4.4% (-17.3%, 8.5%)	0.4599
**Family Size**												
<=6	27.8% (26.8%, 28.7%)	22.2% (21.3%, 23.1%)	-5.6% (-6.9%, -4.3%)	<0.001	34.1% (32.4%, 35.8%)	23.3% (21.9%, 24.6%)	-10.8% (-12.8%, -8.8%)	<0.001	54.9% (39.9%, 69.9%)	17.6% (11.3%, 23.9%)	-37.3% (-52.0%, -22.7%)	0.0003
>6	30.1% (29.2%, 31.0%)	26.8% (25.7%, 27.8%)	-3.3% (-4.7%, -2.0%)	<0.001	40.5% (38.8%, 42.1%)	26.7% (25.4%, 28.0%)	-13.8% (-15.7%, -11.9%)	<0.001	30.9% (16.1%, 45.8%)	31.7% (23.9%, 39.5%)	0.7% (-10.7%, 12.2%)	0.8852

Table S3: Not immunized prevalence and change across equity parameters by region

Equity Parameters	Karachi				KP				Baluchistan			
	BL % (95 % CI)	EL % (95 % CI)	EL-BL % (95 % CI)	P-Values	BL % (95 % CI)	EL % (95 % CI)	EL-BL % (95 % CI)	P-Values	BL % (95 % CI)	EL % (95 % CI)	EL-BL % (95 % CI)	P-Values
**Overall**	15.0% (14.2%, 15.9%)	8.1% (7.4%, 8.8%)	-6.9% (-8.0%, -5.8%)	<0.001	26.1% (24.8%, 27.3%)	14.1% (13.1%, 15.0%)	-12.0% (-13.4%, -10.6%)	<0.001	39.1% (24.4%, 53.7%)	10.9% (6.9%, 15.0%)	-28.1% (-45.3%, -10.9%)	0.0049
**Wealth Quintiles**												
Poorest	20.8% (18.8%, 22.9%)	8.2% (6.7%, 9.8%)	-12.6% (-15.1%, -10.2%)	<0.001	25.1% (22.5%, 27.7%)	13.9% (12.2%, 15.6%)	-11.2% (-14.3%, -8.2%)	<0.001	13.3% (-14.0%, 40.6%)	11.5% (7.4%, 15.6%)	-1.8% (-30.4%, 26.7%)	0.887
Poor	18.1% (16.6%, 19.7%)	7.9% (6.7%, 9.0%)	-10.2% (-12.1%, -8.4%)	<0.001	30.0% (27.9%, 32.1%)	16.8% (15.2%, 18.4%)	-13.2% (-15.7%, -10.7%)	<0.001	50.8% (21.8%, 79.7%)	18.0% (10.7%, 25.2%)	-32.8% (-67.3%, 1.7%)	0.060
Middle	14.7% (13.4%, 16.0%)	8.5% (7.4%, 9.5%)	-6.2% (-7.9%, -4.6%)	<0.001	29.0% (27.1%, 31.0%)	17.1% (15.5%, 18.7%)	-11.9% (-14.2%, -9.6%)	<0.001	54.6% (32.5%, 76.7%)	8.4% (2.6%, 14.2%)	-46.2% (-73.3%, -19.1%)	0.004
Rich	10.7% (9.6%, 11.8%)	7.9% (6.9%, 8.9%)	-2.8% (-4.3%, -1.3%)	0.0002	27.0% (25.1%, 28.8%)	13.5% (12.1%, 15.0%)	-13.4% (-15.6%, -11.2%)	<0.001	37.6% (17.1%, 58.0%)	8.6% (3.5%, 13.8%)	-28.9% (-52.2%, -5.7%)	0.020
Richest	11.2% (9.8%, 12.5%)	8.2% (7.0%, 9.4%)	-3.0% (-4.8%, -1.2%)	0.0010	19.4% (17.5%, 21.4%)	8.8% (7.5%, 10.1%)	-10.6% (-12.8%, -8.4%)	<0.001	37.0% (21.8%, 52.2%)	8.0% (2.5%, 13.4%)	-29.0% (-45.8%, -12.2%)	0.004
**Mother education**												
Literate	5.5% (5.0%, 6.1%)	3.2% (2.7%, 3.6%)	-2.4% (-3.1%, -1.7%)	<0.001	23.7% (22.2%, 25.3%)	10.2% (9.3%, 11.1%)	-13.5% (-15.2%, -11.8%)	<0.001	25.6% (2.8%, 48.3%)	7.4% (-8.3%, 23.1%)	-18.2% (-28.8%, -7.5%)	0.004
Illiterate	24.0% (22.7%, 25.2%)	12.8% (11.7%, 13.9%)	-11.2% (-12.7%, -9.6%)	<0.001	27.9% (26.4%, 29.4%)	18.3% (16.9%, 19.7%)	-9.6% (-11.4%, -7.8%)	<0.001	41.1% (26.3%, 55.8%)	11.1% (7.0%, 15.1%)	-30.0% (-48.0%, -12.0%)	0.004
**Gender**												
Male	14.3% (13.4%, 15.3%)	7.9% (7.1%, 8.6%)	-6.5% (-7.6%, -5.3%)	<0.001	26.0% (24.7%, 27.3%)	13.2% (12.2%, 14.2%)	-12.8% (-14.3%, -11.4%)	<0.001	39.4% (24.6%, 54.2%)	10.2% (6.1%, 14.3%)	-29.2% (-46.3%, -12.1%)	0.004
Female	15.8% (14.8%, 16.7%)	8.4% (7.6%, 9.2%)	-7.4% (-8.6%, -6.2%)	<0.001	26.1% (24.8%, 27.5%)	15.2% (14.2%, 16.3%)	-10.9% (-12.4%, -9.4%)	<0.001	38.7% (24.1%, 53.3%)	11.8% (7.4%, 16.1%)	-27.0% (-44.6%, -9.3%)	0.007
**Age**												
0-23	15.8% (14.8%, 16.7%)	8.9% (8.1%, 9.6%)	-6.9% (-8.1%, -5.7%)	<0.001	27.8% (26.5%, 29.2%)	11.0% (10.2%, 11.9%)	-16.8% (-18.3%, -15.3%)	<0.001	42.5% (27.2%, 57.7%)	12.0% (7.0%, 17.0%)	-30.5% (-48.5%, -12.5%)	0.004
24-59	14.5% (13.6%, 15.4%)	7.7% (6.9%, 8.4%)	-6.9% (-8.0%, -5.7%)	<0.001	25.2% (23.8%, 26.5%)	15.8% (14.7%, 16.9%)	-9.3% (-10.9%, -7.8%)	<0.001	36.7% (21.7%, 51.6%)	9.0% (5.0%, 13.0%)	-27.7% (-45.3%, -10.0%)	0.006
**Family Size**												
<=6	11.2% (10.3%, 12.0%)	5.8% (5.2%, 6.4%)	-5.4% (-6.4%, -4.3%)	<0.001	21.2% (19.8%, 22.7%)	13.0% (11.9%, 14.1%)	-8.3% (-9.9%, -6.6%)	<0.001	19.2% (5.8%, 32.7%)	4.7% (2.4%, 7.1%)	-14.5% (-29.0%, 0.0%)	0.0502
>6	18.3% (17.2%, 19.4%)	11.0% (9.9%, 12.1%)	-7.3% (-8.8%, -5.9%)	<0.001	28.5% (27.0%, 29.9%)	14.6% (13.5%, 15.7%)	-13.9% (-15.5%, -12.2%)	<0.001	43.0% (29.6%, 56.5%)	12.6% (8.2%, 17.1%)	-30.4% (-46.4%, -14.4%)	0.0020

Table S4: Polio zero dose prevalence and change across equity parameters by region

Equity Parameters	Karachi				KP				Baluchistan			
	BL % (95 % CI)	EL % (95 % CI)	EL-BL % (95 % CI)	P-Values	BL % (95 % CI)	EL % (95 % CI)	EL-BL % (95 % CI)	P-Values	BL % (95 % CI)	EL % (95 % CI)	EL-BL % (95 % CI)	P-Values
**Overall**	14.2% (13.4%, 15.1%)	8.0% (7.3%, 8.7%)	-6.2% (-7.3%, -5.2%)	<0.001	35.7% (34.2%, 37.2%)	18.4% (17.3%, 19.5%)	-17.3% (-19.0%, -15.5%)	<0.001	37.9% (22.2%, 53.6%)	13.0% (5.5%, 20.4%)	-24.9% (-45.2%, -4.6%)	0.0215
**Wealth Quintiles**												
Poorest	19.5% (17.5%, 21.5%)	8.8% (7.1%, 10.6%)	-10.7% (-13.2%, -8.1%)	<0.001	41.3% (37.9%, 44.8%)	19.5% (17.6%, 21.5%)	-21.8% (-25.8%, -17.8%)	<0.001	13.3% (-14.0%, 40.6%)	8.5% (4.6%, 12.4%)	-4.8% (-34.0%, 24.4%)	0.717
Poor	17.4% (15.8%, 18.9%)	7.6% (6.4%, 8.7%)	-9.8% (-11.6%, -8.0%)	<0.001	41.6% (39.1%, 44.0%)	21.6% (19.9%, 23.4%)	-19.9% (-22.9%, -17.0%)	<0.001	52.3% (23.1%, 81.5%)	20.3% (9.0%, 31.7%)	-32.0% (-72.0%, 8.1%)	0.104
Middle	13.9% (12.6%, 15.2%)	8.1% (7.1%, 9.1%)	-5.8% (-7.4%, -4.2%)	<0.001	37.9% (35.8%, 40.1%)	21.4% (19.7%, 23.2%)	-16.5% (-19.1%, -13.9%)	<0.001	52.6% (25.8%, 79.5%)	12.2% (3.4%, 21.0%)	-40.4% (-74.3%, -6.6%)	0.024
Rich	9.9% (8.9%, 10.9%)	7.7% (6.7%, 8.7%)	-2.2% (-3.6%, -0.7%)	0.0029	34.7% (32.6%, 36.8%)	17.7% (16.1%, 19.2%)	-17.0% (-19.4%, -14.6%)	<0.001	35.8% (15.6%, 56.1%)	11.3% (5.0%, 17.5%)	-24.6% (-49.1%, -0.1%)	0.050
Richest	10.7% (9.3%, 12.0%)	7.7% (6.5%, 8.9%)	-3.0% (-4.7%, -1.2%)	0.0011	23.6% (21.4%, 25.7%)	11.4% (10.0%, 12.8%)	-12.1% (-14.5%, -9.7%)	<0.001	36.1% (19.8%, 52.3%)	11.5% (1.9%, 21.0%)	-24.6% (-44.4%, -4.7%)	0.021
**Mother education**												
Literate	5.0% (4.5%, 5.6%)	2.9% (2.5%, 3.3%)	-2.1% (-2.8%, -1.5%)	<0.001	34.3% (32.4%, 36.2%)	14.5% (13.4%, 15.6%)	-19.9% (-21.9%, -17.8%)	<0.001	24.2% (0.1%, 48.3%)	9.3% (-8.0%, 26.6%)	-15.0% (-28.5%, -1.4%)	0.034
Illiterate	22.8% (21.6%, 24.0%)	12.8% (11.7%, 13.9%)	-10.0% (-11.6%, -8.5%)	<0.001	36.7% (35.0%, 38.5%)	22.7% (21.2%, 24.2%)	-14.0% (-16.1%, -11.9%)	<0.001	39.9% (24.1%, 55.7%)	13.1% (5.5%, 20.8%)	-26.8% (-48.1%, -5.5%)	0.019
**Gender**												
Male	13.6% (12.6%, 14.5%)	7.8% (7.0%, 8.5%)	-5.8% (-6.9%, -4.6%)	<0.001	36.1% (34.5%, 37.7%)	17.6% (16.5%, 18.7%)	-18.5% (-20.3%, -16.7%)	<0.001	38.1% (22.0%, 54.2%)	12.6% (5.3%, 19.8%)	-25.6% (-45.7%, -5.4%)	0.018
Female	14.9% (14.0%, 15.8%)	8.2% (7.4%, 9.0%)	-6.7% (-7.9%, -5.5%)	<0.001	35.1% (33.6%, 36.7%)	19.5% (18.3%, 20.7%)	-15.6% (-17.4%, -13.8%)	<0.001	37.6% (22.3%, 52.9%)	13.4% (5.5%, 21.4%)	-24.2% (-44.9%, -3.4%)	0.027
**Age**												
0-23	12.7% (11.8%, 13.5%)	6.9% (6.2%, 7.6%)	-5.8% (-6.9%, -4.7%)	<0.001	33.4% (31.9%, 34.9%)	11.5% (10.6%, 12.4%)	-21.9% (-23.6%, -20.2%)	<0.001	37.8% (21.9%, 53.7%)	8.7% (4.0%, 13.5%)	-29.0% (-46.7%, -11.4%)	0.005
24-59	15.3% (14.3%, 16.3%)	8.7% (7.9%, 9.6%)	-6.6% (-7.8%, -5.4%)	<0.001	36.9% (35.3%, 38.5%)	22.4% (21.1%, 23.7%)	-14.5% (-16.4%, -12.6%)	<0.001	37.9% (21.9%, 54.0%)	20.9% (8.7%, 33.0%)	-17.1% (-41.8%, 7.7%)	0.153
**Family Size**												
<=6	10.6% (9.7%, 11.4%)	5.6% (5.0%, 6.3%)	-5.0% (-6.0%, -3.9%)	<0.001	25.0% (23.4%, 26.7%)	16.9% (15.7%, 18.2%)	-8.1% (-10.0%, -6.2%)	<0.001	16.8% (4.5%, 29.1%)	5.1% (1.8%, 8.4%)	-11.7% (-26.0%, 2.6%)	0.0963
>6	17.3% (16.2%, 18.3%)	10.9% (9.8%, 12.0%)	-6.4% (-7.8%, -4.9%)	<0.001	40.9% (39.1%, 42.7%)	19.1% (17.9%, 20.4%)	-21.8% (-23.9%, -19.7%)	<0.001	42.1% (27.3%, 56.9%)	15.1% (6.7%, 23.6%)	-27.0% (-46.5%, -7.5%)	0.0121
